# Editorial: Morphologically Complex Words in the Mind/Brain

**DOI:** 10.3389/fnhum.2016.00047

**Published:** 2016-02-16

**Authors:** Alina Leminen, Minna Lehtonen, Mirjana Bozic, Harald Clahsen

**Affiliations:** ^1^Department of Clinical Medicine, Center of Functionally Integrative Neuroscience and MINDLab, Aarhus UniversityAarhus, Denmark; ^2^Cognitive Brain Research Unit, Cognitive Science, Institute of Behavioural Sciences, University of HelsinkiHelsinki, Finland; ^3^Department of Psychology, Abo Akademi UniversityTurku, Finland; ^4^Department of Psychology, University of CambridgeCambridge, UK; ^5^Potsdam Research Institute for Multilingualism, University of PotsdamPotsdam, Germany

**Keywords:** morphology, derivation, inflection, compound, L2, dyslexia, semantics, decomposition

In most languages, sentences can be broken down into words, which themselves can be further decomposed into units that contain meaning of their own, so-called morphemes (e.g., “*play”* or plural form “-*s*”). Morphemes are the main building blocks and tools, which we use to create and change words. The representation of morphologically complex words (inflected, derived, and compound) in the mental lexicon and their neurocognitive processing has been a vigorously investigated topic in psycholinguistics and the cognitive neuroscience of language. Are morphologically complex words such as “*player*” and “*plays*” decomposed into their constituents (i.e., into their stem “*play*” and plural suffix “-*s*” or agentive suffix “-*er*”) or are they processed and represented holistically (“*player*” and “*plays*”)? Despite extensive research, many important questions remain unanswered. Our Research Topic addresses several currently unresolved topics on the time-course of morphological analysis and the relationship between form and meaning information in morphological parsing. The studies also seek answers to the questions of how inflections and derivations differ in the way they are handled by the mental lexicon, how compound words are recognized and produced, as well as how morphologically complex words are processed within the bilingual mental lexicon, as well as by different clinical populations.

With respect to *time-course of morphological processing* and *interplay between form and meaning*, many current models assume that morphological processing proceeds by analyzing form first at the very earliest stages of processing, after which meaning of the morphemes is accessed (e.g., Rastle and Davis, 2008). In contrast, Feldman et al. provided evidence for the view that meaning information comes into play even at the very early stages of morphologically complex word recognition. Two studies (Estivalet and Meunier; Smolka et al.), focusing on the role of *semantic transparency and regularity* in derived and inflected words indicate decomposition in semantically and phonologically opaque and transparent words in two different languages. That is, both semantically transparent and opaque derivations were found to be represented and processed in similar ways in German (Smolka et al.), and all inflected verbal forms in French showed decomposition effects during visual recognition (Estivalet and Meunier), regardless of their regularity and phonological realization, thus supporting models of obligatory morphological decomposition (e.g., Taft, 2004). Two neuroimaging studies in this Research Topic elucidated the neural correlates of the processing of regular vs. irregular inflection, a highly debated issue. Using time-resolved magnetoencephalography (MEG) with English verbs, Fruchter et al. found priming effects for visually presented irregular stimuli, quite early in the processing, within the left fusiform and inferior temporal regions. The results were interpreted as favoring a single mechanism account of the English past tense, in which even irregulars are decomposed into stems and affixes prior to lexical access (Stockall and Marantz, 2006), as opposed to a dual mechanism model, in which irregulars are recognized as whole forms (e.g., Pinker, 1991). On the other hand, with Russian, a language with very little scrutiny so far and a relatively novel analysis of fMRI functional connectivity, Kireev et al. reported that functional connectivity between the left inferior frontal gyrus (LIFG) and bilateral superior temporal gyri (STG) was significantly greater for regular real verbs than for irregular ones during production. The results shed new light on the functional interplay within the language-processing network and stress the role of functional temporo-frontal connectivity in complex morphological processes. These two studies with arguably different outcomes suggest that the debate on regular vs. irregular form processing continues. They however also point to the potentially critical influences of the processing modality (written vs. spoken) as well as the task (comprehension vs. production) on the mechanism of morphological processing.

Turning to a question of *inflected and derived word processing*, where several previous studies have observed differences in the underpinning neural mechanisms (e.g., Leminen et al.; Leminen et al., 2013; Leminen et al., for a review see e.g., Bozic and Marslen-Wilson, 2010). Service and Maury report differences between derivations and inflections in working memory (as measured by simple and complex span tasks), suggesting different levels of lexical competition and hence, differential lexical storage. Using combined magneto- and electroencephalography (M/EEG), Whiting et al. defined the spatiotemporal patterns of activity that support the recognition of spoken English inflectional and derivational words. Results demonstrated that spoken complex word processing engages the left-hemisphere's fronto-temporal language network, and, importantly, does not require focused attention on the linguistic input (Whiting et al.). Using a similar auditory passive oddball paradigm and EEG, Hanna and Pulvermuller observed that the processing of spoken derived words was governed by a distributed set of bilateral temporo—parietal areas, consistent with the previous literature (Bozic et al., 2013; Leminen et al.). In addition, derived words were found to have full-form memory traces in the neural lexicon (see e.g., Clahsen et al., 2003; Bozic and Marslen-Wilson, 2010; Leminen et al.), activated automatically (see also Leminen et al., 2013).

In the field of cognitive neuroscience of language, a largely under-investigated topic has been the neural processing of *compound words*. An article by Brooks and Cid da Garcia therefore brings an important contribution to elucidating this issue. Their primed word naming task revealed decompositional effects in access to both transparent and opaque compounds. In the MEG results, the left anterior temporal lobe (LATL) as well as the left posterior superior temporal gyrus showed increased activity only for the transparent compounds. These effects were concluded to be related to compositional processes and lexical-semantic retrieval, respectively. Our Research Topic also presents novel findings on written production of compounds, where Bertram et al. introduces an approach rarely used with morphologically complex words. Specifically, they investigated the interplay between central linguistic processing and peripheral motor processes during typewriting. Bertram et al. concluded that compound words seem to be retrieved as whole words before writing is initiated and that linguistic planning is not fully complete before writing, but cascades into the motor execution phase.

With respect to the important topic on *bilingual morphological processing*, our Research Topic presents three studies and one commentary. Lensink et al. used a priming paradigm to show that both transparent (e.g., *moonlight*) and opaque (e.g., *honeymoon*) compounds in the second language (L2) undergo morphological analysis in production. The second study (De Grauwe et al.) used fMRI to assess the processing of Dutch prefixed derived words, demonstrating a priming effect for L2 speakers in the LIFG, an area that has been associated with morphological decomposition. De Grauwe et al. concluded that L2 speakers decompose transparent derived verbs rather than process them holistically. In his commentary on De Grauwe et al.'s article, Jacob discusses the specific aspect of decomposition that the LIFG finding might be reflecting, as well as the extent to which the findings can be generalized to all derivations, instead of one particular verb class. In the third article, Mulder et al. examined the role of orthography and task-related processing mechanisms in the activation of morphologically related complex words during bilingual word processing. Their study shows that the combined morphological family size is a better predictor of reaction times (RTs) than the family size of individual languages. This study also demonstrates that the effect of morphological family size is sensitive to both semantic and orthographic factors, and that it also depends on task demands.

Last but not least, two studies aimed to provide insights into morphological processing by analyzing neglect and letter position issues in dyslexic population. Reznick and Friedmann suggested that the effect of morphology on reading patterns in neglexia provides supportive evidence that morphological decomposition occurs pre-lexically, in an early orthographic-visual analysis stage. Using a different dyslexic population, letter position dyslexics, Friedmann et al. reached a similar conclusion that morphological parsing takes place at an early, pre-lexical stage and that decomposition is structurally rather than lexically driven.

To summarize, this Research Topic presents an overview of a wide range of questions currently addressed in the field of morphological processing. It highlights the significance of morphological information in language processing, both written and spoken, as assessed by the variety of methods and approaches presented here. The partly discrepant findings in some of the contributions to our Research Topic also underline the need for increased cross-talk between researchers using different methods, modalities, and paradigms.

## Author contributions

AL wrote the main paper, ML and MB edited the manuscript, HC provided conceptual advice.

### Conflict of interest statement

The authors declare that the research was conducted in the absence of any commercial or financial relationships that could be construed as a potential conflict of interest.
